# Nepal's War on Human Rights: A summit higher than Everest

**DOI:** 10.1186/1475-9276-4-9

**Published:** 2005-06-28

**Authors:** Sonal Singh, Khagendra Dahal, Edward Mills

**Affiliations:** 1 MPH Student, Bloomberg School of Public Health, Johns Hopkins University, Baltimore, Maryland, USA; 2 Department of Medicine, Unity Health System, Affiliate of the University of Rochester, Rochester, NY, USA; 3 International Student Representative, International Physicians for the Prevention of Nuclear War, Katmandu, Nepal; 4 Centre for International Health and Human Rights, University of Oxford, UK

## Abstract

Nepal has witnessed serious human rights violations including arbitrary arrests, detentions, "disappearances", extra judicial executions, abductions and torture carried out by both the Royal Nepalese Army and the Maoist rebels in the 10 years of the "peoples war". Women and children have borne the brunt of the conflict. Massive displacement has led to adverse social and psychological consequences. While the reasons for the conflict are mainly indigenous and rooted in the social and economic in-equities, remedies for health inequities must come not only from the health sector but also from broad social policies and adopting a participatory and conflict-sensitive approach to development. Meanwhile the international community needs to use its leverage to urge both sides to accept a human rights accord and honor international human rights and humanitarian laws, while investigating allegations of abuse and prosecute those responsible.

## Introduction and recent events

Nepal is a Himalayan kingdom in south Asia, sandwiched between India and China. Serious human rights violations have escalated since the Communist Party of Nepal (Maoists) launched a "people's war," against the government forces in 1996. On February 1^st ^of this year (2005), King Gyanendra of Nepal announced a state of emergency in Nepal, assuming direct rule over the kingdom for a planned 3 years. Political leaders, including the prime minister and opposition leaders, were placed under house arrest. Many student leaders, human right activists and pro-democrats were detained. News media were censored with security personnel patrolling the streets on high alert. According to the king "There is spiraling violence by the rebels which has caused enormous suffering to the people and nation, but the political parties are just fighting among themselves and have been unable to stop it." [1] This dramatic event attempts to strengthen the Royal Nepalese army, an army which has already been accused of serious human rights violations, and has heightened the possibility of further human rights violations in Nepal.

While the reasons for the current conflict are mainly political, we explore some of the social economic and health inequities at the root of the conflict, the current human rights situation in Nepal and its impact on the more vulnerable of the population, mainly women and children. We also explore the role of the international community and developmental and humanitarian Non Governmental Organisations (NGOs) in the current conflict and their successes and failures in promoting the cause of human rights and health equity in Nepal.

## Inequities at the root of the "Peoples War"

As one of the poorest countries in the world, Nepal has a gross national income of US$240 per person [2] and a population of more than 23 million, where some 85 % reside in villages and the majority (82%) survive on less than 2 dollars a day. In the last two and a half decades, the country has experienced an average economic growth rate of four per cent. However, the number of people below the poverty line has doubled from 4.7 million in 1976 to 9 million in 2002.[3]

The indicators of health and quality of living are very meager in this part of the world. Life expectancy at birth is 61 years. The maternal mortality ratio is 539 per 100000 livebirths, [4] and the infant mortality rate is 64 per 1000 live births.[5]

There are widespread disparities in the health care indicators between people from different class and geographical areas. This is evident from the comparison of under-five mortality rates (U5MR) among women on the basis of their education level: U5MR for children of uneducated mothers is 121 per 1000 births which is 64 per cent higher than for children of mothers with primary education, and is nearly double that for children of mothers with secondary level education.[5] Similarly, U5MR in urban areas is 93.6 per 1000, whereas in rural and mountainous regions it increases to 147 and 201 per 1000, respectively. [5] There are also differences in immunization coverage, nutritional status and health care delivery. Similarly, the distribution of the total of 3200 physicians in Nepal is imbalanced: the average physician-patient ratio is 4 doctors per 100,000 people in Nepal that rises to only 1 physician per 150, 000 people in remote hilly areas.[6]

The disparities are also reflected in the Human Development Index (HDI), a composite index of education, health (life expectancy at birth (LEB)) and income, which shows a close association with the caste hierarchy. Brahmans, Newars, and Chhetris, are well above the national average while indigenous people, Dalits (untouchables) and Muslims are below the national average.[7] The marginalization of indigenous people and Dalits, and discrimination along caste and gender lines, with the widening rural-urban divide are a key factor in the current conflict.

Originating in the western heartlands region of Nepal, which has some of the worst indicators, the conflict now has spread to all 75 districts. It has led to widespread disruption of infrastructure and affected the delivery of health services throughout the country.[8] The conflict has claimed more than 11,000 lives and human rights violations have escalated since the collapse of a cease-fire between the two sides in August 2003. Nepal has become a country of human generated disasters [9]. Underdeveloped roads and fragile communication links (only 14 phone lines for 1000 people) [10], in a rugged mountainous terrain suited for guerilla warfare, has allowed both sides to perpetuate crimes against civilians with complete disregard for the rule of law.

## Human rights abuses - disappearances and torture

US based Human Rights Watch claims that both the Maoist rebels and the Royal Nepalese Army are engaged in regular intimidation and extortion leading to a climate of intense fear in Nepal.[10] The government forces have resorted to large-scale arbitrary arrests, detentions, "disappearances", extra judicial executions and torture including rape.[11,12] Human rights defenders, including lawyers; journalists and members of NGO's have been arrested, tortured, killed or "disappeared" in Nepal. [12] Nepal held the unique distinction for the highest number of "disappearances" of any country in 2003 and 2004.[13] The Maoists have resorted to torture and deliberate and unlawful killings.[11,13] According to INSEC (Informal Sector Service Centre), a human rights organisation, nearly 3000 people were killed and about 26,000 people were abducted in 2004 in Nepal.[14] The Maoists have abducted civilians, including teachers and schoolchildren for the purpose of 'political indoctrination'. [13]

More than 70% of Nepalese prisoners claim to have been tortured while in custody [15] The Centre for Victims of Torture, (CVICT) Nepal, an NGO based in Kathmandu claims that some 16,000 people are subject to torture in Nepal every year, affecting an estimated 100,000 people including family members. [16] According to data compiled by CVICT, at the beginning of the Maoist insurgency 80 percent of the victims were subjected to torture from the state and the remainder by the Maoist rebels. However, a recent study in the mid-western district of Jajarkot showed that the number subjected to torture by Maoists had doubled and reached 40 percent [16]. A recent study by Danish researchers confirmed the presence of torture by both the government forces and Maoists in mid-western Nepal[17] and our survey of Tibetan refugees fleeing to Nepal at the Tibetan Refugee Transit Centre in Kathmandu confirmed the presence of torture by both the security forces and the rebels.[18] The long-term repercussions of torture on health and psychological well-being are considered devastating. [19]

## Role of health professionals

Some physicians have contributed to the politics of Nepal in the last decades in the struggle to ensure health as a human right within the broader macroeconomic and political picture.[20] The government has prosecuted physicians for the ethical practice of providing care for those injured, including rebels, violating international ethical standards set by the World Medical Association. [21] The government has issued directives to all health professionals and institutions stating that if health professionals provide treatment without appropriate notification, they will be regarded as supporters of terrorists and be prosecuted according to the Terrorist and Disruptive Activities Ordinance, 2001.[22] The directive was outlined by the Ministry of Health in a public meeting: "doctors working both in government hospitals and private health institutions are liable to government action if they treat terrorists without getting permission from the security wings... if any doctor defies, action will be taken against him or her as per the recently promulgated ordinance against terrorists".[22] This directive puts medical professionals in an impossible situation: during the ongoing conflict, medical professionals are at risk of encountering armed groups demanding treatment for their wounded; however, provision of such treatment might lead to subsequent government prosecution. [21] The Nepalese Medical Council (NMC), the only national body ensuring medical ethics, has remained silent on this issue.

## Consequences of conflict

Nepal has witnessed a gradual increase in the incidence of depression, posttraumatic stress disorder and suicide since the beginning of the conflict [23]. Mental health services, which were rudimentary to begin with, have been further fragmented. Health experts estimate the prevalence of mental health problems in Nepal to be as high as 30 %. [23]

Women and children are bearing the major brunt of the war. With literacy among women as low as 36%, [24] the political violence has had a negative impact on women's rights and health.[25] The Maoists have capitalized on the plight of women, who have been marginalized for decades in Nepalese society and enrolled them into the conflict in large numbers [26] Nearly half of all Maoist rebels are women and their sexual exploitation is not uncommon. The conflict has also contributed to an increase in the trafficking of Nepalese women and girls, nearly 5000 to 10,000 a year to Indian brothels. [27] The youth have fled the country in large numbers to Indian cities and the Middle East, leaving women and children behind.

Children have been particularly affected by the insurgency. [28] Some estimate that around 100, 000 children have been affected by the war and the numbers likely to increase to 500,000 as the conflict expands.[29] Conservative estimates in 2003 showed that at least 146 children have died, 2000 have been orphaned, and 3000 have become homeless.[29] While the government vehemently denies the use of child-soldiers, around 10–15% of the recruits are under the age of 18 years (possibly due to birth registration irregularities which are not uncommon in developing nations).[30] The Maoists previously denied the use of child-soldiers. However, according to an estimate in 2000, around 30 % of the Maoist soldiers were children.[31] They have been utilized as informants, porters, and for cultural propaganda. Earlier in 2004 the Maoists announced a plan to create a militia of 50,000 child soldiers.[32] Although the numbers of recruits planned may be ambitiously inflated, they have resorted to mass- abduction of children as young as 12 from schools and classrooms in Western Nepal. The abducted children are indoctrinated and given training in guerilla warfare.[32] This marks a major departure from their previous commitments to avoid recruiting children below the age of 18.

## Refugee crisis

Nepal provides support to nearly 100,000 Bhutanese refugees under the aegis of the United Nation High Commission for Refugees (UNHCR). The refugee crisis has been compounded by the fact that many have moved within Nepal as a result of the conflict between Maoists and the government. Most realistic estimates put their number between 100,000 and 200,000. [33] The displaced Nepalese have either flocked to the main cities or fled the conflict to India. Displacement of the Nepalese – population has given rise to social problems commonplace amongst migrant populations. The government, to a large extent, has ignored the plight of internally displaced persons (IDPs). [33]

## Human rights conventions being flouted

The civil war in Nepal meets the Geneva Conventions definition of an internal armed conflict. [34,10] The Maoist rebels have an identifiable and organized command structure, both at the national and regional level, are in de-facto control of a significant part of Nepali territory [10]. Both the government of Nepal, which ratified the Geneva Conventions in 1964, and the Maoist rebels have agreed to abide by them. [10] One of the most fundamental protections during internal armed conflicts is contained in Article Three common to the four Geneva Conventions of 1949. This governs the treatment of civilians and captured combatants during internal armed conflicts, and outlaws summary executions, torture and other ill treatment of persons, the taking of hostages, and punishment without fair trial. This has been violated by both parties to the conflict.

In addition to the laws of war, the government of Nepal is a party to all the major human rights treaties, including the International Covenant on Civil and Political Rights (ICCPR),[35] which it acceded to in 1991, and the Convention against Torture and Other Cruel, Inhuman or Degrading Treatment or Punishment.[36] which prohibits arbitrary arrest and detention, torture and other mistreatment, enforced disappearances, and extrajudicial executions. Efforts initiated by the National Human Rights Commission of Nepal, established in 1999, to have a human rights accord signed have failed. In Jan 2005 the United Nations Human Rights Commissioner; Louis Arbour criticized both the governments and the Maoist leaders in Nepal for not doing enough to tackle human rights violations. [37] The reason for the human rights abuses has been the impunity enjoyed by the security forces under the Terrorist and Disruptive practices Act (TADA). [38]

Political paralysis with dozens of failed governments in the last decade and a crumbling monarchy that witnessed fratricide, patricide and matricide in one chilling day 4 years ago have meant a loss of confidence by the Nepalese people in the political process.[39] And, the recent political turnover has made the situation worse by suspending the fundamental rights to freedom, expression, information, property and free travel. Human rights abuses are increasing as a result of the new regime's deliberate involvement in creating armed militias and other vigilante groups throughout the country encouraging them to conduct offensive attacks against civilians in the name of resisting the Maoists. [40]

## Role of the international community

The international community is sharply divided between supporting the Nepalese army, with a dubious record of abuses on one hand, and the brutal Maoist rebel movement on the other. Previously, the US, India and the United Kingdom had supported the Nepalese government with weapons; the US supplying US$29 million US in military aid from 2001 to 2004, [10]largely viewing the Maoist problem as a part of its global "war on terror". On the other hand, the European Union and the United Nations have condemned both sides for human rights abuses. No government has supported the Maoists. Although Nepal's conflict is mainly internal and will require indigenous solutions addressing decades of poverty and inequality, with antagonistic and uncompromising political visions, it is difficult to envisage a solution without the intervention of a third party. It is imperative that the international donor community, which provides for nearly 60 % of Nepal's development budget, particularly the states most active in Nepal – India, the U.S., the U.K. and the European Union – should act decisively and in concert to promote adherence to international human rights and humanitarian law in Nepal. The recent political developments in Nepal have led to a temporary curb in military and developmental aid to Nepal by several countries, although it is unlikely that the government will cut down on military spending, thus developmental projects in the rural areas might be the hardest hit.

## Role of humanitarian and development NGOs and conflict sensitive development

While Nepal is flooded with NGOs, and most of them are developmental NGOs paradoxically, development assistance may have unknowingly exacerbated the conflict by perpetuating the same inequalities, which led to the conflict in the first place. Many international agencies have inadvertently contributed to the conflict by raising the expectations of the rural poor. NGO projects have mainly benefited the urban majority while the rural minority still suffers in poverty.[41] Foreign aid, which accounts for nearly 60 % of Nepals developmental budget, may have paradoxically contributed to lopsided development in Nepal. While aid money has favored urban development the rural-urban gap has widened over the years. In Nepal, weak linkages between urban and rural areas and lack of roads, communications, infrastructure and appropriate skills among the rural poor mean that this urban bias has led to of centralization of effective power on the one hand, and maintenance of the economic, social and political status quo, on the other hand. The Maoists have forced several international agencies to leave remote western regions, where help is needed the most, while the government has put several administrative roadblocks in the way of international agencies working in Nepal.[42] Save the Children's work in the Accham district of western Nepal has been hindered by fighting between the Maoist rebels and government forces since early 2002 [43]. Offices of non-governmental organizations (NGOs) have been burnt and volunteers are afraid to work.[43] Even Médecins Sans Frontières (MSF) – not a development NGO but a humanitarian NGO – was forced to curtail its activities last year in Jumla, one of the poorest districts in Midwest, due to the conflict. In May 2005 four international agencies the World Food Programme, Britain's DFID and German GTZ and Dutch SNV aid agencies suspended their program in western Nepal as the rebels attacked aid-workers. [44]

In the context of Nepal – as in many other wars – a more nuanced approach to humanitarian relief and protection and development agendas would be helpful-one that recognizes a clear distinction between humanitarian relief and development. They are not the same and should not be lumped together. The distinction is critical in that it can mean the difference between the relief of the immediate suffering of war, or not. Humanitarian relief is to be given in a manner consistent with universal medical ethics; on the basis of need alone; impartially, with the giver as a neutral agent between the parties to the conflict (with crimes against humanity, war crimes, genocide and egregious violations of international humanitarian law being the outer limit of neutrality), and in a fashion that is independent of government, rebel or third party interests. Meeting all these criteria can conflict with a development agenda that seeks to overcome structural inequities that can be both cause and the conditions of war. It is not that inequities must not nor cannot be addressed, just not in a structural manner by actors who operate under the aegis of humanitarianism in war. To do so is to effectively seek to rewrite public policy and practice, which are in many cases in war, contested and a principle issue of contention in the conflict. To seek to do this is to effectively engage the politics of the particular war, which necessarily means taking sides – sometimes different sides at different times, but taking sides none-the-less, thus endangering the direct provision of humanitarian relief and protection in war. It is not possible – practically or politically – for an agency – NGO or otherwise – to pursue a development agenda in a war while simultaneously providing humanitarian relief.

Development NGOs in Nepal may consider pursueing an approach of "conflict-sensitive development"[44] – development sensitive to the (conflict) environments in which NGOs operate, in order to reduce the negative impacts of their activities – and to increase their positive impacts – on the situation and its dynamics. Development projects can continue in less affected areas with a need for transitional programs in conflict areas that can adapt to the rapidly changing environment. Some agencies have adopted a participatory role in development and have involved neutral local agencies, increasing community participation in their projects with good success. There is also a need for increasing coordination between organizations working in various projects.[45] Multicultural, multilingual and multiethnic representation and participation are essential components in the design of any successful developmental programmes in Nepal. Remedies for health inequities must come not only from the health sector but also from broad social policies that address potential health gaps related to equity e.g. distribution of income. Reducing illiteracy might significantly decrease the vulnerability of women to the effects of other health risks.

## Recommendations

There is a need to provide immediate assistance to internally displaced persons; protect the independence and effectiveness of the judiciary; ensure the continued independence, institutional continuity and stability of the NHRC; and ensure the full and unimpeded access of the NHRC, the office of the High Commissioner of Human Rights (OHCHR) and the International Committee of the Red Cross to all detention centers, including Royal Nepal Army barracks. [10] The Nepalese justice system, which lacks independence, training and resources, must step up to the challenge. The OHCHR and other internationally recognized organizations must embark on continuing education programmes and intensive human rights education for Nepalese judges and prosecutors. The Nepalese government and army must allow such persons to carry out their duties without pressure or threats. [46]

It is necessary that both sides comply with international human rights and humanitarian law, in particular prohibitions on attacks on civilians; executing or ill-treating persons in custody; committing "disappearances," abductions and unlawful arrests; and committing acts of extortion or looting.[10] There is a need to investigate all allegations of abuse and appropriately prosecute the perpetrators in accordance with international fair trial standards. There is also an urgent need to implement a human rights accord, which abides by the Geneva conventions and commit both the government and Maoists to abide by clear human rights standards and accept human rights monitoring. This Accord was drawn up by the NHRC and widely promoted by the international and human rights community, including by the High Commissioner for Human Rights during her visit to Nepal in January 2005. The Accord would be a valuable confidence building measure towards future peace negotiations.

The recent establishment of an Office of the High Commissioner for Human Rights in Nepal is an important step towards protecting human rights in Nepal. [47]. Under the April 10 Memorandum of Understanding signed between the government of Nepal and the U.N. High Commissioner for Human Rights the mandate of the mission is to "monitor the observance of human rights and international humanitarian law, bearing in mind the climate of violence and the internal armed conflict in the country. [48] If the mission is to succeed, this mandate must be interpreted broadly to cover all aspects of human rights violations, and not be restricted to reporting on specific cases. If investigations are limited to specific incidents of torture, killing and abduction, the non-functioning of judicial institutions as a primary cause of these abuses will be missed. The U.N. mission will have to engage closely with local people and sympathetic and interested Nepalese, both inside and outside the country. While both sides have welcomed the UN monitoring of human rights in Nepal it will remain to be seen whether this leads to an improvement in the human rights situation. The difficult geographical terrain of Nepal and limited communication links makes the process of human rights monitoring a major challenge. Although the agreement is clear, the international community must remain vigilant to ensure that this agreement is complied with effectively and fully

The international community was effective in reducing the number of disappearances last year and will have to use its leverage in reducing the number of killings. Given the importance that Nepal places on its international image and its dependence on international assistance, the position that the international community adopts will be of critical importance in the coming months. It is therefore important that the international community, when sending a strong message about the importance of restoring democracy, stresses that this must be a democracy with human rights and protection for a pluralist civil society at its core. [49] Failure to address these issues at this critical hour runs the risk of leaving Nepal on a slippery slope of chaos and anarchy. In the meantime, the "People of Nepal", in whose name the war is being fought, will continue to be its main casualty, as they face renewed threats of violence, displacement and hunger with every passing day.
